# *GNQ-209P* Mutation in Metastatic Uveal Melanoma and Treatment Outcome

**DOI:** 10.1155/2018/4256365

**Published:** 2018-04-04

**Authors:** Nagla Abdel Karim, Ihab Eldessouki, Ahmad Taftaf, Deeb Ayham, Ola Gaber, Abouelmagd Makramalla, Zelia M. Correa

**Affiliations:** ^1^Department of Hematology-Oncology, University of Cincinnati, Cincinnati, OH, USA; ^2^Department of Interventional Radiology, University of Cincinnati, Cincinnati, OH, USA; ^3^Department of Ophthalmology, University of Cincinnati, Cincinnati, OH, USA

## Abstract

Metastatic prognosis in uveal melanoma is assessed by gene expression profiling (GEP) testing of the tumor cells, usually obtained by fine needle aspiration (FNA). GEP has demonstrated high accuracy in distinguishing class I and II tumors, both having different metastatic potential. Transcriptomic studies identified distinct mutations including somatic mutations in *GNAQ* and *GNA11*, detected in more than 80%, and contribute to the upregulation of the mitogen-activated protein kinase (MAPK) pathway and the development of uveal melanoma (UM). The role of these mutations in treatment selection and possible benefit from targeted therapy are somewhat unclear. However, until the discovery of novel agents, local versus systemic therapies remain options for treatment that can still be considered for disease control in certain cases. We report a series of patients with metastatic UM with distinct mutational profiles. One had significant liver metastases with proven *GNQ-209P* mutation on tissue biopsy while peripheral blood molecular profiling did not show these mutations. The other three cases had no *GNQ-209P* mutation. All cases received nab-paclitaxel (Abraxane) as a treatment drug, and we record their responses to treatment and their molecular-profiling results.

## 1. Introduction

Uveal melanoma (UM) is significantly less common than cutaneous melanoma and has a distinct molecular pathogenesis. Meanwhile, it is the most common primary intraocular tumor in adults [1]. Despite the high success rate of disease control with local therapy, the potential for developing metastases remains high even after a prolonged period of remission [2–4]. The predominant target organ for metastasis is the liver although disease involvement of the skin, bone, brain, and lungs has also been reported [5, 6]. Key mutations in the disease are *GNAQ* and *GNA11* mutations. It was reported that 83% of the cases have somatic mutations in *GNAQ* or *GNA11* [7]. *GNAQ* gene is the gene coding for the alpha subunit of heterotrimeric G proteins. The latter proteins couple seven-transmembrane domain receptors to intracellular signaling machinery [8], and they are composed of three subunits, namely, alpha, beta, and gamma. The alpha subunit is the G-protein molecular switch, activated when it is bound to guanosine triphosphate (GTP), and when GTP is hydrolyzed to guanosine diphosphate (GDP), it is deactivated [9]. The alpha subunit has a key glutamine that contacts the GTP molecule, located at position 209 (Q209) in G*α*q and is substituted when mutated to either leucine or proline [10–13]. At this point, the alpha subunit is locked in a constitutively active state, and its GTPase activity is blocked [14–16]. Taxanes work by preventing microtubule disassembly, so the mitotic functions are inhibited, leading to cell death [17]. They have shown reasonable activity in several phase II studies [18]. Nab-paclitaxel is a solvent-free formula that renders the drug more competent in the treatment of UM. Multiple therapeutic approaches for metastatic UM have been studied although none has shown any impact on the overall survival, and thus standard of care has not yet been established for these patients. In our report, we present the case of a patient with metastatic uveal carcinoma to the liver who was successfully treated with nab-paclitaxel, allowing for recovery from life-threatening spontaneous tumor lysis.

## 2. Methods

*GNAQ* and *GNA11* mutations were assessed by genomic hybridization on paraffin-embedded blocks obtained from the primary tumor tissue. Mutations were followed up by circulating tumor DNA in plasma using next-generation sequencing using serial blood samples.

## 3. Case Presentation

**Figure 1:**
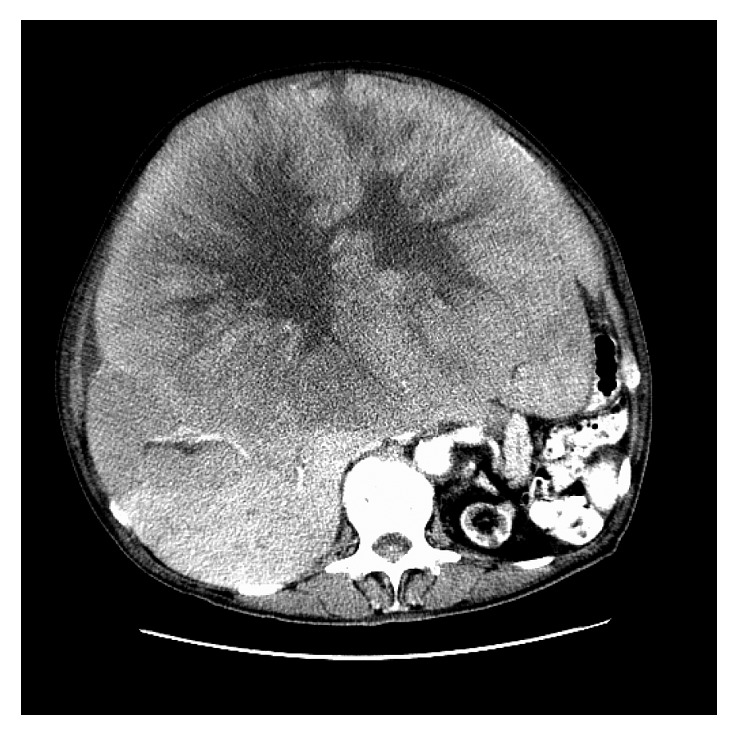
Abdominal CT scan at diagnosis.

**Figure 2:**
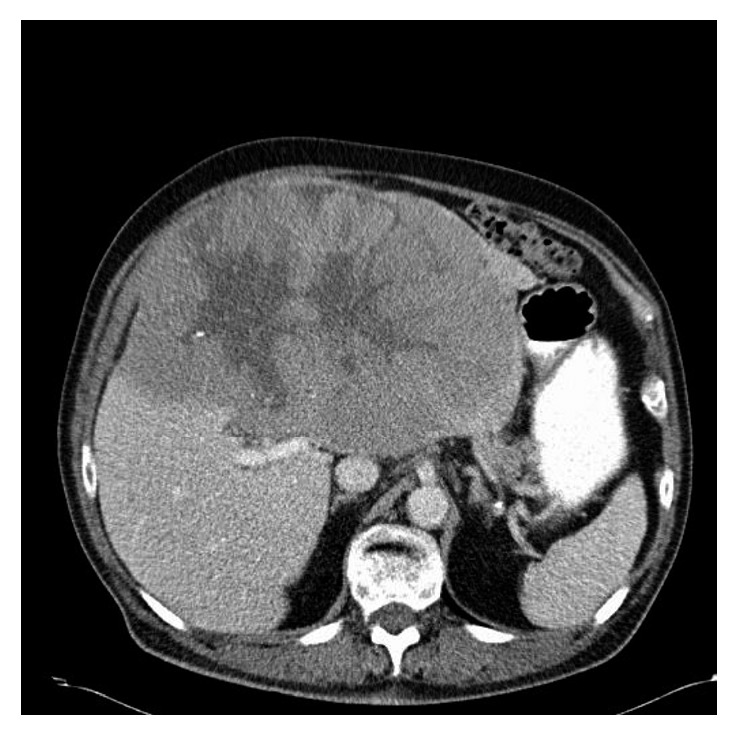
CT scan 24 months after the treatment.

This is a 75-year-old man with a history of choroidal melanoma of the right eye diagnosed in 1984 and treated by radioactive Co-60 plaque. Thirty years later, he presented with progressive abdominal distention, early satiety, and weight loss of 20 pounds over a period of 6 months. He was seen by his primary care physician who requested a CT scan of the abdomen that showed a large hepatic mass measuring 34 cm by 26 cm, replacing the majority of the liver without retroperitoneal or mesenteric lymphadenopathy (Figure 1). Hepatic tumor biopsy revealed metastatic melanoma consistent with his primary choroidal melanoma. While completing his diagnostic workup, the patient developed generalized weakness prompting his hospital admission due to acute renal failure, hyperkalemia, and spontaneous tumor lysis. He started hemodialysis promptly followed by the administration of weekly nab-paclitaxel 150 mg/m^2^ and then reduced to 75 mg/m^2^ thereafter due to severe neutropenia. The patient recovered his renal function as serum creatinine improved from 4.93 mg/dl to 0.69 mg/dl (normal values 0.60–1.20 mg/dl) and demonstrated clinical improvement of his generalized weakness, abdominal distention, and edema of the legs after three doses of nab-paclitaxel. A repeat abdominal CT scan one month after the therapy revealed a good response to treatment with significant decrease in tumor burden. This is donated by full clinical recovery and total resolution of tumor lysis manifestations. However, according to RECIST criteria, the response can be minimal followed by maintained stable disease. CT scan of the abdomen after 4 cycles of nab-paclitaxel revealed shrinkage of the hepatic lesion to 24 × 15 cm in maximum diameter (approximately 7% decrease in the largest lesion per RECIST criteria) (Figure 2). This patient is still alive and continues to have excellent functional status, ECOG performance status of I, and no signs or symptoms of disease progression for 32 months now.

**Figure 3:**
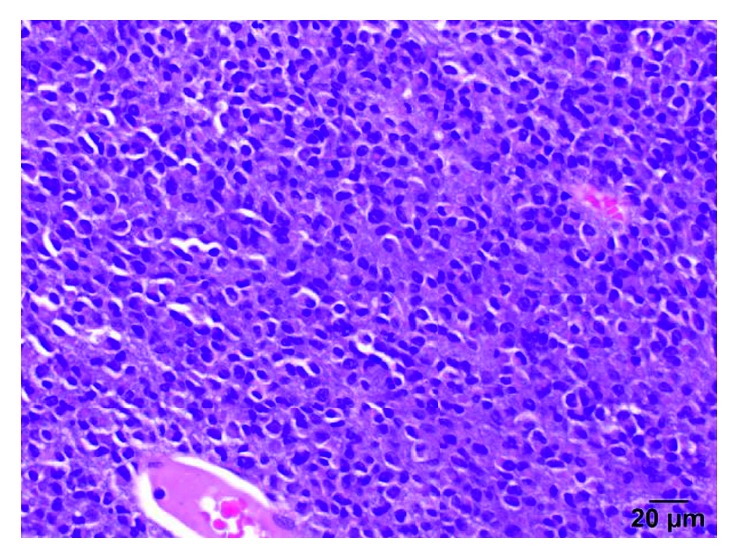
Liver biopsy (H&E).

**Figure 4:**
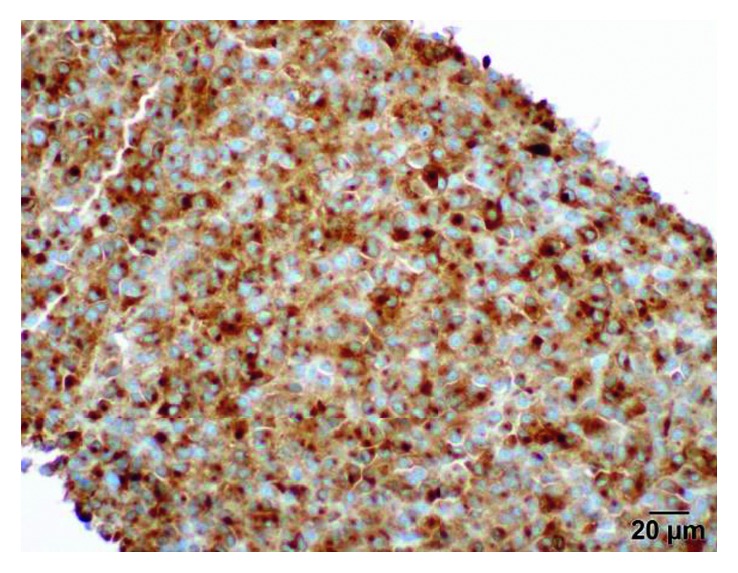
Liver biopsy (MART).

Our patient with this metastatic uveal melanoma with extensive liver metastases with *GNQ-209P* mutation on the tissue biopsy (Figures 3 and 4) and undetectable mutations on the peripheral blood molecular profiling in serial follow-up samples suggests marked response to nab-paclitaxel. This can be understood by the dramatic tumor response on CT scans which was accompanied clinically by spontaneous tumor lysis syndrome followed by very prolonged disease control up to 30 months indicating nab-paclitaxel efficacy. All other patients with metastatic ocular melanoma, who did not have the *GNQ-209P* mutation, did not respond and did not have prolonged survival when treated with nab-paclitaxel.

Our patient has received 8 cycles of Abraxane with initial minimal response followed by no increase and stable tumor size in the following imaging scans. In an attempt to achieve further response, the patient received an anti-PD-L1 in a clinical trial for 9 cycles. No further reduction in tumor size was achieved, and the patient was disqualified from the study after he developed sarcoidosis/interstitial pneumonitis. He was then restarted on Abraxane, achieving clinical and radiological stabilization of his disease with no major toxicities, and remains fully functional. He has received to date 12 cycles of Abraxane (in addition to the prior cycles of Abraxane received initially).

## 4. Discussion

UM has a high potential for developing a rapidly progressive course despite local or systemic therapies [1]. Even with several FDA-approved agents for advanced cutaneous melanoma, there is a lack of agents that show survival benefit in patients with advanced UM. This issue is likely twofold from the rare occurrence of the disease itself as well as a lack of complete understanding regarding the pathogenesis and immunobiology that underlies this disease process. Current studies are ongoing to uncover these uncertainties in hopes of ultimately identifying potentially treatable targets and more effective treatments [19, 20]. Until a standard of care is established, however, existing treatment options must be applied on a case-by-case basis [21, 22].

In metastatic disease, different approaches have been studied including surgical resection in suitable candidates in addition to local versus systemic infusion of cytotoxic agents. A comprehensive review of the role of metastectomy in selected surgical candidates showed improved survival in patients who had a complete liver metastases resection compared to patients for whom a complete resection was not feasible [23]. Local therapies including the hepatic arterial infusion of melphalan or fotemustine revealed in randomized trial, a significant improvement in progression-free survival (PFS) but not overall survival when compared to the systemic infusion of the same agents [24]. In contrast, a retrospective study from Mayo Clinic showed only improvement in overall survival among patients treated with different local therapies in comparison to different systemic agents including bevacizumab, ipilimumab, and kinase inhibitors [25]. Systemic chemotherapy options have shown minimal benefit in treatment. Single agents such as cisplatin or paclitaxel versus combined agents such as the BOLD regimen (bleomycin + vincristine + lomustine + dacarbazine) plus recombinant interferon alpha 2-b have been studied with no more than 20% response rate (RR) and absence of survival advantages [7, 26]. A similar study to ours that was presented at the ASCO annual meeting shows clinically useful responses in two of four patients with metastatic ocular melanoma treated with nab-paclitaxel [27]. In terms of targeted therapy, the ability to understand the genetic characteristic of UM has helped in identifying different mutations and key signaling pathways that can permit therapeutic intervention at a site specific to the pathway abnormality. UM is genetically characterized by frequent, mutually exclusive mutations in guanine nucleotide-binding protein G(q) subunit alpha (*GNAQ*) and guanine nucleotide-binding protein subunit alpha-11 (*GNA11*) which can be detected in 83% of patients with UM [12]. *GNAQ* stimulates the mitogen-activated protein kinase (MAPK), which is parallel to the consequence of mutations in the BRAF or NRAS oncogenes in cutaneous melanomas. Furthermore, *GNAQ* stimulates the transcriptional coactivator YAP that is essential for UM cell proliferation. The aforementioned MAPK pathway is highly interconnected with the PI3K/ACT pathway, and both of them converge on the same downstream targets. MEK inhibitor, PI3K inhibitor, mTOR inhibitor, and YAP inhibitor each represent novel therapeutic target for UM, and studies are ongoing to uncover the role of these agents either as a single or dual inhibition approach in patients with advanced disease or early disease associated with high-risk features [10, 20, 28].

Our patient presented with acute renal failure secondary to spontaneous tumor lysis, and there was an urgent need for disease control, which was achieved by using systemic chemotherapy with nab-paclitaxel. The absence of standard of care in these patients and the extrapolated data from the phase III trial of nab-paclitaxel when compared to dacarbazine [29] in patients with cutaneous melanoma led to the use of this agent in our patient who was not a candidate for cisplatin. In his case, the tissue from the liver biopsy was insufficient to run molecular testing, so we used a liquid biopsy (circulating tumor DNA) obtained from the patient, to search for genomic alterations that came back negative for mutations; however, subsequent liver biopsy revealed *GNAQ* exon 5 Q209 mutation where the *GNA11* mutation or amplification was not detected.

In comparison to this case (Case 1), we had other patients (Case 2, 3, and 4) in our institution, in whom a diagnosis of metastatic UM was made. All patients started on treatment with nab-paclitaxel, but they had metastatic disease that continued to progress. In patients (Cases 2 and 3), a molecular testing of DNA circulating in the blood revealed *GNAQ*/Q209L mutation while our patient (Case 1) had *GNAQ*-Q209P (Tables 1 and 2). This might draw our attention that *GNQ-209P* might be a predictive marker of sensitivity to nab-paclitaxel in metastatic uveal melanoma.

## 5. Conclusion

Although our understanding of the molecular underpinnings of UM continues to improve and certain targeted agents are showing promise, genomic alteration studies might play a role in treatment selection. As we see from the cases we present, *GNAQ*-Q209P and not Q209L mutation could be associated with a considerable disease control when treated with nab-paclitaxel chemotherapy. We suggest the implication of molecular profiling with specific attention to the status of not only *GNAQ* but also the exon 209P or Q209L for personalized use of therapies in future clinical trials designed to treat patients with metastatic UM. Such clinical trials are needed to prove the efficacy and survival advantages of nab-paclitaxel in patients with metastatic UM and to study its role in comparison to the evolving targeted or immunotherapeutic agents. In general, reports of rare and less commonly encountered cases can have a pivotal effect on the collective clinical experience and drug research.

## Figures and Tables

**Table 1:** Different types of genomic mutations in patients with metastatic melanoma of the liver.

Case number	Age (years)	Gender	Genomic mutation
1	75	Male	*GNAQ*-Q209P by tissue testing
2	77	Female	*GNAQ*-Q209L by peripheral circulating DNA
3	70	Female	*GNAQ*-Q209L
4	78	Female	Kit H580y

**Table 2:** Comparison between *GNAQ*-Q209P and *GNAQ*-Q209L.

Mutations	Location of mutation	Frequency of mutations among *GNAQ*-mutated primary uveal melanoma
*GNAQ*-Q209P	Exon 5	∼64%
*GNAQ*-Q209L	Exon 5	∼33%
